# Development of a Wideband Precision Electric Field Measuring Sensor

**DOI:** 10.3390/s23239409

**Published:** 2023-11-25

**Authors:** Zhaozhi Long, Feng Zhou, Fuchang Lin, Jiawei Fan, Wenting Li, Yinglong Diao, Kangmin Hu

**Affiliations:** 1 School of Electrical and Electronic Engineering, Huazhong University of Science and Technology, Wuhan 430074, China; fclin@hust.edu.cn; 2 China Electric Power Research Institute, Beijing 100192, Chinafanjw@epri.sgcc.com.cn (J.F.); liwenting@epri.sgcc.com.cn (W.L.); diaoyinglong@epri.sgcc.com.cn (Y.D.);

**Keywords:** electric field measuring sensor (EFMS), nonlinearity, impulse high voltage, uniformity coefficient, calibration

## Abstract

High-voltage electric field measurement technology has certain applications in electric field measurement of power systems, but due to the limitation of its measurement accuracy and bandwidth, it cannot be used for the measurement of lightning-impulse voltage. In order to calibrate the nonlinearity of the MV-level lightning-impulse voltage measurement system, this paper proposes the design and implementation of a high-precision inductive wideband electric field measuring sensor (EFMS). The influence of the metal shell on the electric field distribution was simulated, and the influence of the electric field non-uniformity coefficient was studied. The characteristics of the EFMS were tested, and the results showed that the EFMS can accurately reproduce the waveform of lightning-impulse voltage and power-frequency voltage, with a proportionality coefficient of 0.05664 V/(kV/m). In mostly uniform and extremely non-uniform fields, the nonlinearity of the EFMS for impulse voltage is less than ±0.25%, and the nonlinearity of the EFMS for power-frequency voltage is less than 0.1%. It is shown that the EFMS can be used for the nonlinearity calibration of ultra-high voltage impulse measurement devices.

## 1. Introduction

The accuracy of the measurement of lightning-impulse voltage directly affects the evaluation of the insulation withstand ability of electrical equipment and the effectiveness of the insulation distance in transmission lines [1,2]. With the continuous increase in transmission voltage worldwide, the requirements for the insulation level of transformers and transmission equipment have also increased. The shock voltage threshold of 1000 kV electrical equipment can reach ±2400 kV, so most power research institutions and manufacturers are equipped with 3600 kV lightning-impulse voltage test facilities. In China, the ultra-high voltage AC research base has established a ±7500 kV lightning-impulse voltage test facility for the study of long gap discharge.

The standard lightning-impulse voltage measurement system, which converts the measured value into national or international standard, is the most accurate impulse voltage measurement method at present. However, the rated voltage of the internationally recognized standard impulse voltage measurement system is generally only 1500 kV [3,4,5], which cannot meet the requirements of full-scale calibration. Additional linear calibration tests are required for the impulse voltage measurement system to obtain an accurate scaling factor. Therefore, the evaluation of the nonlinearity of the high-voltage part of the high-voltage impact measurement device is a key aspect of the lightning-impulse voltage measurement technology [6,7,8,9].

The development of a standard measuring device for linear calibration tests becomes more difficult with the increase in the rated voltage of lightning voltage [10,11]. High-voltage electric field measurement technology is widely used in power-system power-frequency electric field and voltage measurements due to its advantages of non-contact connection, small volume, and not limited by rated voltage [12,13,14,15,16].

According to different principles, an electric field measuring sensor (EFMS) can be divided into two types [15,17,18]: electric field induction and electro-optic effect [16,17]. The electric field induction type has good measurement stability, but the electric field under test conditions is easily affected by the metal shell. Electro-optical effect type has a sensor head without metal or nonlinear electronic components, which has little influence on the electric field under test conditions, but it is greatly affected by the ambient temperature, and its stability needs to be improved [19,20].

Electric field measurement in power systems has a relatively low requirement for measurement error, and the frequency band width of the waveform to be measured is relatively narrow. Due to the limitations of measurement accuracy and bandwidth, the existing electric field measuring device cannot accurately measure lightning-impulse voltage [21,22].

In order to meet the requirements of measurement accuracy of impulse voltage, in-depth research on sensor structure, material, and signal processing algorithm is needed to improve the measurement accuracy and measurement bandwidth of EFMSes [23,24].

In order to realize the calibration of nonlinearity of an MV-level impulse voltage measurement system, a high-precision broadband EFMS based on electric field induction is developed in this paper, which is used for measuring power frequency, high-frequency AC, and impulse voltage. Firstly, the measurement principle of the broadband EFMS is analyzed, and a design method of the broadband EFMS based on electric field induction is proposed. Secondly, the influence of the metal shell on the electric field distribution is simulated, and the requirement of the non-uniformity coefficient is put forward. The characteristic test platform of the broadband EFMS is built, and the scaling factor and nonlinearity of the power-frequency electric field and the impulse electric field are obtained by comparison method. Finally, the typical application scenarios of broadband EFMS are studied.

## 2. Measuring Principle

### 2.1. Sensing Principle

**Figure 1:**
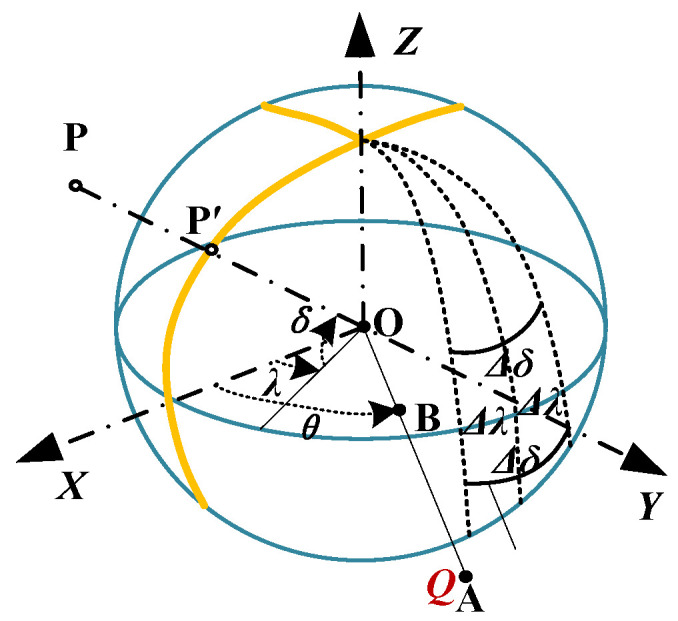
Schematic diagram of probe principle.

The point charge electric field is used as the field source. In actual measurements, because of the small voltage induced between the spherical shells, spherical probes can be approximated as an equipotential body. As shown in Figure 1, *Q* is the point charge, the *X*O*Y* plane is where the field source is located, the *Z*-axis is parallel to the direction of the probe, and the radius of the spherical shell is *R*.

We define the center of the probe to be located at the coordinate origin, *θ*, as the angle to the axis in the XOY plane, and point A (*d*sin*θ*, *d*cos*θ*, 0) at a distance from the origin *d*(*d* > *R*) with a charge amount of *Q*. We estimate that the spherical shell is an equipotential body. Furthermore, using the mirror principle, a small charge is seen at point B (*R*^2^/*d*sin*θ*, *R*^2^/*d*cos*θ*, 0) in the spherical shell, with *q* = −(*R*/*d*)·*Q* as the mirror image of *Q*. The other mirror charge is at O, and the charge quantity is *q*′ = *q* = −(*R*/*d*)·*Q*. Take any point P outside the sphere with coordinates of (*r*cos*δ*cos*λ*, *r*cos*δ*sin*λ*, *r*sin*δ*), and use the potential at infinity as the reference point to obtain the potential at P, which can be represented by Equations (1)–(3) [12]:

(1) $$\varphi_{1}=\frac{Q}{4\pi \varepsilon \bar{\mathrm{AP}}}=\frac{Q}{4\pi \varepsilon \sqrt{r^{2}+d^{2}-2rd\cos\delta \cos(\theta -\lambda )}}$$

(2) $$\varphi_{2}=\frac{q^{\prime }}{4\pi \varepsilon \bar{\mathrm{OP}}}=\frac{RQ}{4\pi \varepsilon rd}$$

(3) $$\varphi_{3}=\frac{Q}{4\pi \varepsilon \bar{\mathrm{BP}}}=\frac{-RQ}{4\pi \varepsilon \sqrt{R^{4}+r^{2}d^{2}-2R^{2}rd\cos\delta \cos(\theta -\lambda )}}$$

where *ε* is the permittivity of space; *R* is the radius; *r* is the distance from point O to point P; *d* is the distance from O to Q; *δ*, *γ*, and *θ* are used to represent the coordinates of any point in a spherical coordinate system. $\bar{\mathrm{BP}}$, $\bar{\mathrm{OP}}$, and $\bar{\mathrm{AP}}$ represent the distance between two points. *φ*_1_, *φ*_2_, and *φ*_3_ represent the potentials generated by different charges at point P.

Let P′ represent the intersection point between the line connecting points P and O and the sphere, with P being any arbitrary point. Thus, P′ can be any point located on the sphere. Considering that the electric field on the sphere is solely characterized by its normal component, the electric field intensity at point P′ can be described using Equation (4):

(4) $$E_{p^{\prime }}=-\frac{\partial \phi_{p}}{\partial r}\left|{}_{r=R}\right.=-\frac{Q}{4\pi \varepsilon ad^{2}}\left\{\frac{1-a^{2}}{1+a^{2}-2a\cos\theta \cos{\left(\theta -\lambda \right)}^{\frac{3}{2}}}-1\right\}$$

where *a* = *R*/*d* is the inhomogeneity coefficient, the surface charge density at point P′ (i.e., the density of any point on the spherical surface), which can be represented using Equations (5) and (6):

(5) $$\sigma_{P^{\prime }}=\varepsilon E_{P^{\prime }}=-\frac{\varepsilon E_{0}}{a}=\left\{\frac{1-a^{2}}{1+a^{2}-2a\cos\delta \cos{\left(\theta -\lambda \right)}^{\frac{3}{2}}}-1\right\}$$

(6) $$E_{0}=\frac{Q}{4\pi \varepsilon d^{2}}$$

where *E*_0_ is the intensity of the electric field at the center of the point to be measured before the sensor is placed.

**Figure 2:**
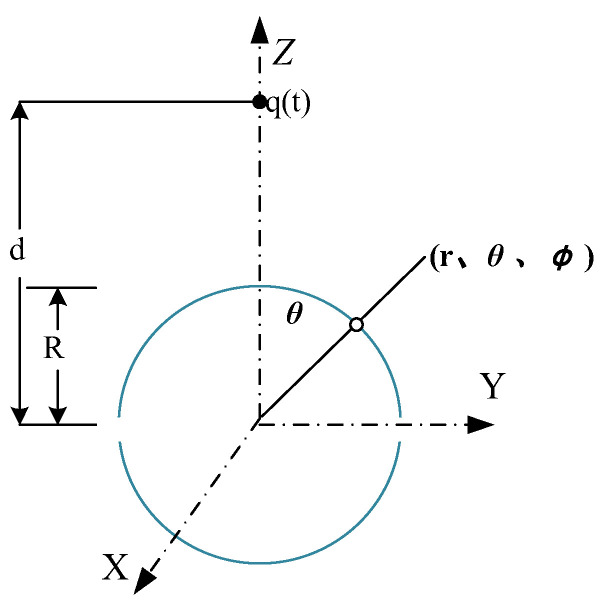
Schematic diagram of measurement principle for one-dimensional spherical electric field probe.

As shown in Figure 2, the amount of charge *Q*_s_ on sphere *S* can be defined as in Equation (7):

(7) $$Q_{s}=-\varepsilon E_{0}\frac{R^{2}}{2a}=\int_{-\Delta \lambda }^{\Delta \lambda }\int_{-\Delta \lambda }^{\Delta \delta }\left\{\frac{1-a^{2}}{1+a^{2}-2a\cos\delta \cos{\left(\theta -\lambda \right)}^{\frac{3}{2}}}-1\right\}\cos\delta d\delta d\lambda$$

When *d* >> *R*, the probe can be considered under the action of a uniform electric field, and parameter *a* is infinitely close to zero. Thus, the charge can be obtained from Equation (8):

(8) $$Q_{s}=-\underset{a\to 0}{\lim}\varepsilon_{0}E_{0}\frac{R^{2}}{2a}=\int_{-\Delta \lambda }^{\Delta \lambda }\int_{-\Delta \lambda }^{\Delta \delta }\left\{\frac{1-a^{2}}{1+a^{2}-2a\cos\delta \cos{\left(\theta -\lambda \right)}^{\frac{3}{2}}}-1\right\}\cos\delta d\delta d\lambda$$

Under the uniform electric field, the induced charge on the sphere *S* can be simplified as Equation (9):

(9) $$Q_{s}=-3\varepsilon E_{0}(t)R^{2}[2\Delta \delta +\sin2\Delta \delta ]\sin\Delta \lambda \cos\theta$$

When splitting the sphere into a one-dimensional spherical probe, Δ*δ* = Δ*λ* = π/2 is obtained from Equation (10), yielding the following:

(10) $$Q_{h}^{\prime }=-3\varepsilon_{0}E_{0}R^{2}\pi \cos\theta =-K^{\prime }E_{0}$$

where *K*′ = −3*εR*2πcos*θ*, $Q_{h}^{\prime }$ represents the induced charge.

The induced voltage across the probe can be represented by Equation (11):

(11) $$U^{\prime }=\frac{Q_{h}^{\prime }}{C}=\frac{K^{\prime }}{C}E_{0}$$

where *C* is the sampling capacitance.

### 2.2. Discussion of Inhomogeneity Coefficients

#### 2.2.1. Theoretical Calculations

In the actual measurement, the electric field probe cannot be placed in an ideal uniform electric field. The difference in the output voltages between electric field probes in uniform and non-uniform fields is analyzed below. For a spherical, one-dimensional electric field probe in the spherical coordinate system, the point charge *q*(t) is the field source and above the *Z*-axis. The spherical center of the probe is located at the coordinate origin O, as shown in Figure 2. The electric field intensity at point O before placing the probe is calculated using Equation (8).

When the power line of the field source *q*(t) coincides with the probe’s measurement direction, the surface charge density of the probe as a function of time can be derived from the previous section as follows:

(12) $$\sigma_{P^{\prime }}(t)=\varepsilon E_{P^{\prime }}{}_{n}(t)=-\frac{\varepsilon E_{0}(t)}{a}=\left\{\frac{1-a^{2}}{{\left(1+a^{2}-2a\cos\theta \right)}^{\frac{3}{2}}}-1\right\}$$

where *R* is the sphere’s radius, and *r* and *θ* are the coordinates in the spherical coordinate system of any point.

When *a →* 0, that is, *R →* 0 or *d →* ∞, the electric field on the probe surface is a nearly uniform field and, from Equation (13), the surface charge density *σ*_1_(*t*) of the probe can be determined:

(13) $$\sigma_{1}(t)=-3\varepsilon E_{0}(t)\cos\theta$$

The total charge of the probe hemisphere under a uniform electric field can be calculated as follows:

(14) Q1(t)=−∫02π∫0π23εE0(t)cosθR2sinθdθdϕ=−3πR2εE0(t)

When *a* ≠ 0, the total charge of the probe hemisphere *Q*_2_(*t*) under the action of a non-uniform electric field can be calculated:

(15) Q2(t)=−∫02π∫0π2σP′(t)R2sinθdθdφ

Since $\left|a^{2}-2a\cos\theta \right|$ < 1, the formula can be expanded into a power series of *a*^2^ − 2*a*cos*θ*. According to the integration of Equation (17) term by term, it can be found that

(16) $$Q_{2}(t)=-3\pi R^{2}\varepsilon E_{0}(t)\left[1-\frac{7}{12}a^{2}+\frac{11}{24}a^{4}-\cdots \right]$$

*C*_m_ represents the sampling capacitance between the upper and lower electrodes of the spherical probe, and the voltage *U*_m_(*t*) at both ends of the capacitor can be represented by Equation (17).

(17) $$U_{m}(t)=\frac{Q(t)}{C_{m}}$$

In the uniform and non-electric fields, the measured voltages *U*_m1_(*t*) can be found using Equations (18) and (19), respectively:

(18) $$U_{m1}(t)=\frac{3\pi R^{2}\varepsilon E_{0}(t)}{C_{m}}$$

(19) $$U_{m2}(t)=\frac{3\pi R^{2}\varepsilon E_{0}(t)\left[1-\frac{7}{12}a^{2}+\frac{11}{24}a^{4}-\cdots \right]}{C_{m}}$$

Therefore, if the EFMS with the proportionality coefficient calibrated in the uniform electric field is used in the non-uniform electric field, the deviation of the proportionality coefficient is shown in Equation (20):

(20) $$\left|\Delta e\right|=\left|\frac{U_{m2}(t)-U_{m1}(t)}{U_{m1}(t)}\right|=\left|-\frac{7}{12}a^{2}+\frac{11}{24}a^{4}-\cdots \right|$$

Based on the above analysis, the measured electric field intensity *E*_0_(*t*) is directly proportional to the induced voltage *U_m_*(*t*) in both uniform and non-uniform fields. However, a difference is present between the proportionality coefficients of uniform and non-uniform electric fields. This difference decreases as the non-uniformity coefficient decreases. When the non-uniformity coefficient *a* is ≤0.1, the deviation in the proportionality coefficient is less than 1%. Therefore, the EFMS using the proportionality coefficient needs to be calibrated in real time.

It can be seen from the above analysis that, in both uniform and non-uniform fields, the measured electric field intensity *E*_0_(*t*) is proportional to the induced voltage *U_m_*(*t*), but there is a deviation in the proportionality coefficient of the EFMS in both uniform and non-uniform fields. With the decrease in the non-uniform coefficient *a*, the deviation of the proportionality coefficient decreases. When the non-uniform coefficient *a* ≤ 0.1, the deviation of the proportionality coefficient is less than 1%. Therefore, it is necessary to carry out real-time calibration of the proportionality coefficient of the EFMS.

#### 2.2.2. Influence of the EFMS on the Measured Electric Field

The EFMS was placed in the measured electric field. The induced charge on the spherical shell was then distorted, and the influence of the EFMS on the measured electric field was simulated and studied.

**Figure 3:**
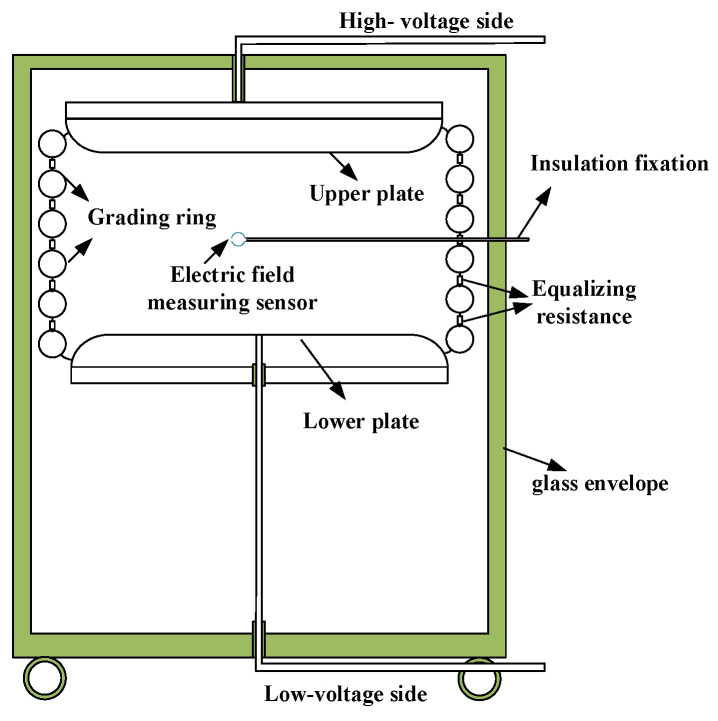
Structure diagram of uniform field generator.

Figure 3 shows the construction of the uniform field generator, in which 12 grading rings realize voltage equalization. The upper and lower plates had a diameter measuring 1.6 m, with a gap of 1 m between them while they were subjected to a voltage of 30 kV.

**Figure 4:**
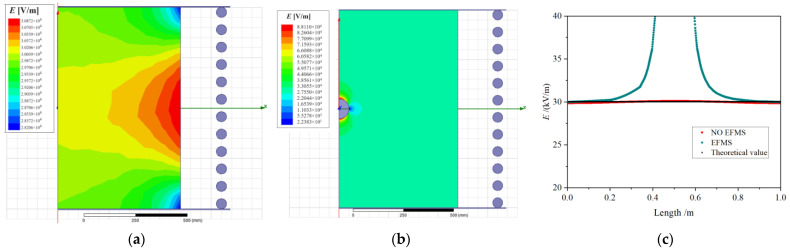
The influence of the EFMS on the measured electric field: (**a**) uniform electric field cloud map; (**b**) the influence of the EFMS on electric field distribution; and (**c**) electric field distribution on the central axis.

Figure 4 shows the results using Ansoft Maxwell 16 electromagnetic simulation software. Figure 4a illustrates the middle part of the electric field cloud map, showing the central electric field intensity of the longitudinal plate slightly higher than the two ends. An EFMS with a diameter of 100 mm was placed at the center between the two plates, and the electric field distribution between the plates is shown in Figure 4b. The electric field intensity increased in the smaller areas above and below the EFMS and was maximized on the spherical surface.

Figure 4c shows the electric field distribution on the central axis when no objects were between the plates; the electric field intensity was very close to the theoretical calculation value, 30 kV/m near the center point. However, when an EFMS was placed in the center, the electric field intensity in that region increased significantly. Within 0.2 m of the upper and lower electrode plates, the deviation between the actual and theoretical electric field intensity values was within 1%, which means that the EFMS did not significantly interfere with the charge distribution on the electrode surface. Changing the conditions with further simulations indicated that, as the EFMS radius decreased, the distance between the EFMS and the measured voltage distribution plate increased, and thus, the influence on the measured electric field reduced.

Further simulations under various conditions indicate that two methods can reduce the influence of the equipotential spherical shell of the EFMS on the measured electric field. One method is to minimize the volume of the EFMS, but excessively small volume may make it challenging to achieve the measurement function. The other method is to increase the distance between the EFMS and the charges. However, as the distance increases, the intensity of the measured electric field decreases, leading to increased interference.

## 3. Design and Development of the EFMS

### 3.1. Structure Design

**Figure 5:**
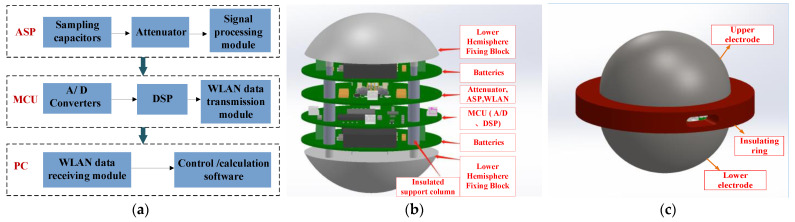
Internal design of field measurement instrument: (**a**) block diagram; (**b**) internal layered design; and (**c**) external structure.

The wideband EFMS comprised an electric field probe, local area network data transmission, and a data reception and processing unit, as shown in Figure 5a. The electric field probe consisted of an upper electrode, an organic glass insulation block, a lower electrode, an analog signal processing module (ASP), an MCU data microprocessor, a WLAN data transmission module, lithium batteries, a WLAN data reception unit, and a PC. The ASP comprised sampling capacitors and an attenuator.

To reduce the size of the EFMS, its internal structure adopted a layered modular design. The sampling rate of the A/D converter was either 10 MS/s or 150 MS/s based on the waveform of the measured voltage. The control software for data processing, acquisition, and transmission was developed using C++. Wireless communication was utilized for data transmission, including an internal WLAN module within the sphere and a data reception module on the PC side.

The recorded data were displayed on the waveform display interface and then connected to the waveform measurement software for waveform parameter calculations. Additionally, the high-speed acquisition and rapid transmission of large data made reducing the overall system’s power consumption challenging. Therefore, rechargeable lithium batteries with high energy density, strong endurance, and small volume were chosen as the power supply. Figure 5b illustrates the internal structure of the EFMS and Figure 5c shows the external structure of the EFMS.

### 3.2. Development of EFMS

Table 1 presents the key technical parameters of the EFMS. The input impedance of the A/D converter was 1 MΩ//35 pF, with a range of ±2.5 V and a vertical resolution of 12 bits. There were two options for the sampling rate: 10 and 150 MS/s. For power-frequency electric field measurements, the sampling rate of the A/D converter was set to 10 MS/s, with a maximum recording length of 1 s. For impulse electric field measurements, it was set to 150 MS/s, with a maximum recording length of 0.2 ms. A wireless local area network was used for the data transmission, with a transmission rate exceeding 2 Mbps. The transmission distance of the WLAN was greater than 30 m.

**Figure 6:**
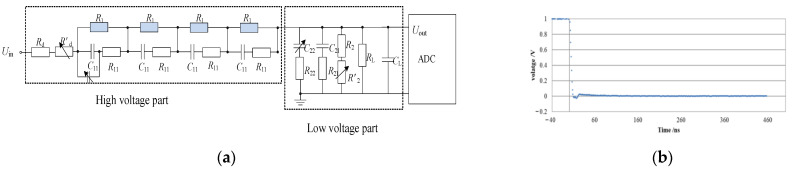
Principle and response of the attenuator. (**a**) Schematic diagram of the attenuator; (**b**) step response waveform.

Due to the small input voltage of the A/D converter, with a maximum range of ±2.5 V, it is necessary to add an attenuator at the front end of the A/D converter. This attenuator scales down the voltage on the sampling capacitor proportionally to adapt to different ranges of electric field intensity measurements. To measure different voltage ranges, the attenuation ratio could be set at 1:1 or 10:1. The schematic diagram of the attenuator is shown in Figure 6a. Due to the influence of distributed capacitance in the loop, parameter matching between the resistance branch and the capacitance branch is a key issue in improving the high-frequency response characteristics of the attenuator. The voltage of the sampling capacitor first enters the waveform processing circuit and the A/D converter through the attenuator. Therefore, the high-frequency response characteristics of the attenuator directly affect the reproduction of the electric field measurement instrument for the transient voltage waveform. A small resistor–capacitor attenuation network was established, with a response time of less than 10 ns, resulting in a significant increase in the measurement bandwidth of the instrument. The step response waveform of the attenuator is shown in Figure 6b.

## 4. Experiment on EFMS Characteristics

### 4.1. Measurement of Power-Frequency Electric Field

**Figure 7:**
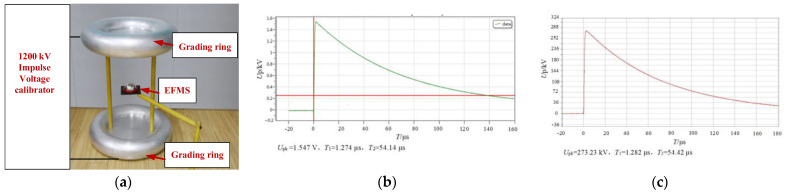
Measurement setting of impulse electric field and measurement results: (**a**) measurement setting; (**b**) waveform of the EFMS; (**c**) waveform of standard resistor divider.

The EFMS was subjected to measurements of the power-frequency electric field in an electromagnetic environment laboratory. First, the spherical electric field probe was positioned at the central location of the electric field source using an insulated support groove. Altering the distance between the upper and lower electrode plates facilitated control over the magnitude of the electric field intensity. A uniform grading ring, similarly designed to that presented in Figure 7a, was implemented between the electrode plates to enforce the equidistribution of the electric field, thereby achieving constancy of the internal spatial field configuration.

The proportionality coefficient of the EFMS was calibrated using the electric field generator shown in Figure 3. Under the condition of an attenuation ratio of 10:1, the proportionality coefficient is the ratio between the output voltage and the measured electric field intensity. Table 2 presents the calibration results for the 50 kV/m to 180 kV/m power-frequency electric field. According to the table, the proportionality coefficient of the EFMS ranges from 0.5658 V/(kV/m) to 0.5665 V/(kV/m), demonstrating high stability. Therefore, under high-frequency voltage conditions, the nonlinearity of the measuring instrument is determined to be 0.08%.

### 4.2. Measurement of Impulse Electric Field

A mostly uniform impulse electric field was constructed using two grading rings, as illustrated in Figure 7a. This configuration entailed two internally flat-plated grading rings. The upper grading ring was linked to a high-voltage lead, while the lower grading ring was grounded. The inter-plate spacing within the circular inner region of both upper and lower grading rings measured 1.5 m. The uniformity coefficient was 0.067. The EFMS apparatus was affixed at the central points along the vertical and horizontal axes of the upper and lower electrode plates.

The waveform of the impulse voltage for the EFMS is shown in Figure 7b,c. The EFMS possesses the capability to measure lightning-impulse voltage. Table 3 shows the comparison of the measurement results between the EFMS and the standard resistor voltage divider. Compared with the standard waveform, the proportionality coefficient for the EFMS was 0.05664. The front time error was −0.6%, and the time to half-value error was −0.5%.

The nonlinearity of the EFMS was calibrated using a 500 kV standard resistor voltage divider (model RS500) with a nonlinearity less than 0.2%. A 10:1 pre-attenuator was set up. The nonlinearity of the EFMS was calibrated in two environments: mostly uniform field and extremely non-uniform field.

For the mostly uniform field, two grading rings with a diameter of 1.5 m were used. The distance between the two electrodes was set to 1 m. The upper electrode was fixed using a crane without the intermediate insulating support rod. The calibration was performed by placing EFMS under the grading rings, approximately 1 m away from them. The calibration voltage ranged from 50 kV to 450 kV. The calibration results are shown in Table 4. From this table, it can be seen that the nonlinearity of the EFMS tends to be consistent in mostly uniform field and extremely non-uniform fields, and the proportion coefficient increases with the increase in the electric field. The relative deviation between the proportion coefficient and the average value of the proportion coefficient under different electric fields is within ±0.25%.

**Figure 8:**
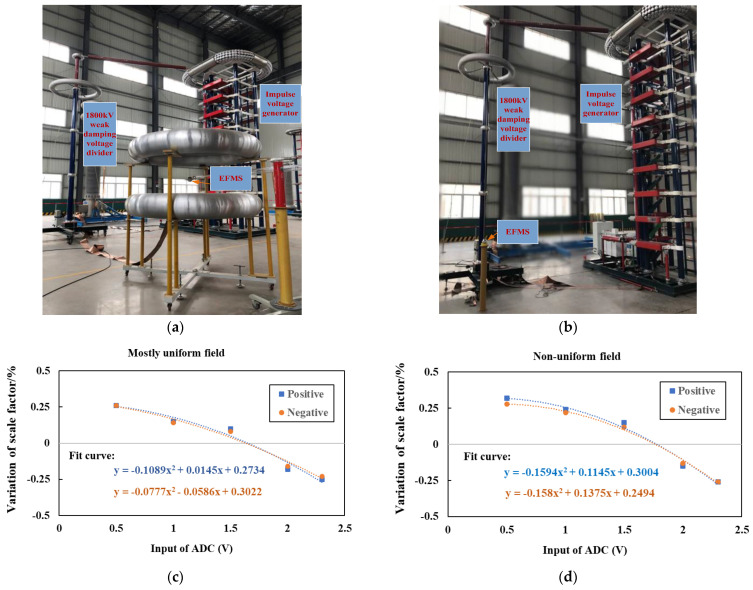
Nonlinearity of the EFMS (attenuation ratio 10:1): (**a**) mostly uniform fields; (**b**) extremely non-uniform fields; (**c**) nonlinearity measurement results in a mostly uniform field; and (**d**) nonlinearity measurement results in an extremely non-uniform field. (The gray line represents the horizontal coordinate axis).

Figure 8 shows the nonlinearity calibration test setting and calibration results for the EFMS. The nonlinearity of the EFMS in extremely non-uniform and mostly uniform fields were calibrated in turn. Figure 8a,b depict the experimental setup for the test.

Figure 8c,d show the nonlinearity test results of the EFMS proportionality coefficient. As can be seen form the figure, the nonlinearity of the EFMS tended to be consistent in extremely non-uniform and mostly uniform field, and the EFMS proportionality coefficient changes within ±0.25% in the ADC range of 0.5 V~2.5 V.

**Figure 9:**
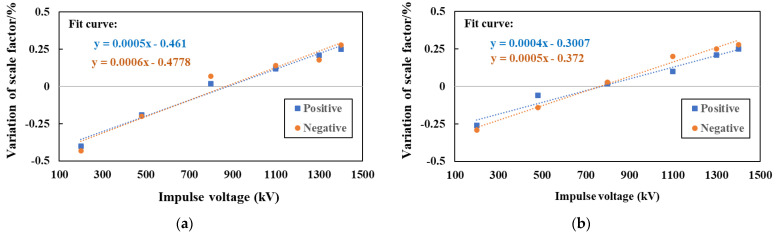
Linearity calibration results of the 1800 kV weakly damped voltage divider: (**a**) the EFMS as a reference standard; and (**b**) the standard resistor voltage divider as a reference standard. (The gray line represents the horizontal coordinate axis).

The nonlinearity of the 1800 kV impulse voltage divider was evaluated using the EFMS developed in this study. During the experiment, the distance between the electrodes was 3.2 m, and the applied impulse voltages were 200 kV, 400 kV, 600 kV, 800 kV, 1000 kV, 1200 kV, and 1400 kV. The experimental results were corrected based on the inherent nonlinearity of the EFMS to obtain the nonlinearity results of the 1800 kV divider, as shown in Figure 9a. Figure 9b shows the nonlinearity evaluation results, which were obtained using a standard impulse divider as a reference standard. As indicated, the broadband EFMS could calibrate the nonlinearity of the high-voltage impulse divider.

## 5. Discussion

This study aimed to investigate the effectiveness of EFMSes in calibrating the nonlinearity of high-voltage dividers. Our findings revealed the potential of EFMSes as reliable calibration tools in high-voltage measurement applications. In this discussion, we elaborate on these findings, compare them with existing calibration methods, analyze their impact, and propose future research directions.

First, our experimental results demonstrated that EFMSes can effectively measure and characterize the nonlinearity of high-voltage dividers. By comparing the readings of EFMSes with the expected voltage values, we observed deviations within an acceptable range for accurate measurements. This indicates that EFMSes hold promise as reliable calibration devices for evaluating the linearity of high-voltage dividers. Notably, EFMSes offer the advantage of not being limited by the measured voltage range, making them versatile tools for calibrating high-voltage dividers of different ranges.

Comparing our findings with existing high-voltage divider calibration methods, such as generator voltage ratio comparison, ratio measurements, and laser interferometry, several advantages of EFMSes become evident. First, EFMSes employ a non-contact measurement technique, reducing dependencies on the performance characteristics of the generator. This ensures a more independent and reliable calibration process. Second, EFMSes offer wide bandwidth and high sensitivity, allowing for accurate measurement of dynamic voltage changes and transient behavior. These advantages position EFMSes as viable options for calibrating linearity in high-voltage measurement scenarios.

However, it is important to note that several factors can affect the accuracy of calibration results based on EFMSes. One critical factor is the distance between the sensor and the grading ring of the divider, along with sufficiently small non-uniformity coefficients, meaning that the sensor diameter should be significantly smaller than the distance to the grading ring. Additionally, the stability of the sensor’s position and its proximity to other charged objects should be considered. Furthermore, real-time calibration is necessary for each measurement when using EFMSes for calibration. While this requirement adds complexity and time to the calibration process compared to some other methods, it ensures accurate and reliable results.

The use of EFMS developed in this paper in a strong electric field will actually cause electric field distortion near the tips of the upper and lower hemispheres, resulting in inaccurate measurement results. Therefore, it is very important to properly position the spherical electric field measuring instrument during the linearity calibration of the high-voltage divider. Generally, the height of voltage dividers exceeding 2 MV is above 8 m. During the measurement process, we increased the distance between the electric field measuring instrument and the grading ring to ensure that the electric field was less than 450 kV/m. From the experimental results, it can be seen that within this range, there will be no sudden change in the output voltage of the electric field measuring instrument. While limiting the electric field value, the electric field measuring instrument will be affected by spatial interference of the electric field.

Another limitation of EFMS-based calibration is its sensitivity to corona discharge and surrounding electric field interference. These factors can introduce measurement errors and affect the calibration outcome. Therefore, employing appropriate shielding techniques and keeping the sensor isolated from external electric fields are crucial for achieving accurate calibration results.

To address these limitations and explore future research opportunities, we propose the following directions. Firstly, conducting additional experiments covering a wider range of high voltages and different types of high-voltage dividers would help establish the capabilities and limitations of EFMS-based calibration methods more comprehensively. Secondly, further research should focus on developing advanced signal processing techniques and algorithms to enhance the accuracy and precision of EFMS measurements while mitigating the impact of corona discharge and interference.

In conclusion, this study showcased the potential of EFMSes as effective tools for calibrating the linearity of high-voltage dividers. The results contribute to the literature by highlighting the advantages of EFMS in high-voltage measurement applications, particularly their versatility across different voltage ranges. Considering the influencing factors and addressing the identified limitations are critical for successful implementation of EFMS-based calibration methods. Future research endeavors should align with the aforementioned research directions to optimize the calibration process and improve overall accuracy and reliability in high-voltage measurements.

## 6. Conclusions

To accurately evaluate the nonlinearity of a multi-MV impulse voltage measurement device, this paper proposes the development and design of a broadband EFMS. The output voltage of the EFMS is linearly related to the measured electric field, and the influence of the EFMS on the measured electric field is analyzed.

To reduce the size, a layered modular design method was used for the internal structure of the EFMS spherical shell to achieve signal processing, analog-to-digital conversion, and data transmission functions. Measurement bandwidth extension techniques and stability improvement methods were studied.

An experimental platform for measuring power frequency and impulse voltage was established, and the calibration and nonlinearity test of the EFMS scale factor under power frequency and impulse voltage was conducted. The test results show that the nonlinearity of the EFMS in an impulse electric field is less than ±0.25%, with an electric field ranging from 50 kV/m to 450 kV/m. The nonlinearity of the EFMS is less than 0.1% in a power-frequency electric field, with an electric field ranging from 50 kV/m to 180 kV/m. Finally, the nonlinearity of a 1800 kV impulse divider in an extremely non-uniform field was evaluated using the EFMS. The evaluation results are consistent with the comparison results of a standard impulse voltage divider. Therefore, it is concluded that the developed EFMS can be used for linearity calibration of ultra-high voltage impulse dividers.

## Figures and Tables

**Table 1:** Main parameters of the EFMS.

Parameters	Value	Parameters	Value
Diameter of the spherical shell	10 cm	Material of the spherical shell	Stainless steel
Input impedance	1 MΩ//35 pF	Range of ADC	±2.5 V
Vertical resolution	12 Bit	Sampling rate	10 and 150 MS/s
Wireless transmission distance	>30 m	Transmission rate	2 Mps
Attenuator	1:1, 10:1, 100:1	Sampling capacitance *C*_m_	3.32 nF

**Table 2:** Power-frequency electric field calibration results.

Electric Field	Output Voltage of EFMS	Proportionality Coefficient	Average
kV/m	V	V/(kV/m)	V/(kV/m)
50	0.2832	0.05663	0.05662
80	0.4526	0.05658	
110	0.6228	0.05662	
140	0.7925	0.05661	
180	1.0197	0.05665	

**Table 3:** Comparison between lightning impulse measurement of the EFMS and impulse voltage divider.

	Peak Voltage (V)	Front Time (μs)	Time to Half-Value (μs)
EFMS	1.547	1.274	54.14
Standard divider	273.23 × 10^3^	1.282	54.42
Error	N/A	−0.6%	−0.5%

**Table 4:** Comparison of electric field measuring instruments results.

Mostly Uniform Field				Extremely Non-Uniform Field			
Electric Field kV/m	Output Voltage of EFMS V	Proportionality Coefficient	Relative Deviation from the Average Value %	Electric Field kV/m	Output Voltage of EFMS V	Proportionality Coefficient	Relative Deviation from the Average Value %
50.1	0.283115	0.05651	−0.22	49.2	246.492	0.05643	−0.23
100.6	0.569396	0.05660	−0.06	100.4	1010.024	0.05649	−0.12
200.1	1.133567	0.05665	0.02	199.4	3989.994	0.05662	0.02
301.2	1.707804	0.05670	0.11	299.1	9008.892	0.05669	0.11
432.6	2.456735	0.05679	0.15	425.4	18402.8	0.05676	0.22
